# Host genetics and gut microbiota composition: Baseline gut microbiota composition as a possible prognostic factor for the severity of COVID-19 in patients with familial Mediterranean fever disease

**DOI:** 10.3389/fmicb.2023.1107485

**Published:** 2023-03-30

**Authors:** Vardan Tsaturyan, Anahit Manvelyan, Marine Balayan, Natalya Harutyunyan, Elya Pepoyan, Tamas Torok, Michael Chikindas, Astghik Pepoyan

**Affiliations:** ^1^Faculty of Military Medicine, Yerevan State Medical University, Yerevan, Armenia; ^2^International Association for Human and Animals Health Improvement, Yerevan, Armenia; ^3^Division of Food Safety and Biotechnology, Armenian National Agrarian University, Yerevan, Armenia; ^4^Earth Sciences Division, Lawrence Berkeley National Laboratory, Berkeley, CA, United States; ^5^Health Promoting Naturals Laboratory, Rutgers State University, New Brunswick, NJ, United States; ^6^The International Scientific-Educational Center of the National Academy of Sciences of the Republic of Armenia, Yerevan, Armenia

**Keywords:** gut microbiota, familial Mediterranean fever disease, COVID-19, disease severity analysis, gender

## Abstract

**Background:**

It is known that the gut microbiome of a healthy person affects the process of COVID-19 after getting infected with SARS-CoV-2 virus. It is also believed that colchicine can alleviate the severity of COVID-19.

**Objective:**

Current investigations aimed to evaluate the associations between the baseline gut microbiota composition of healthy and Familial Mediterranean fever (FMF) - carrier Armenian men populations, and the severity of the COVID-19 disease after their infection with the SARS-CoV-2. The study has a purpose of answering three core questions: i. Do the characteristics of gut microbiome of Armenians affect the course of COVID-19 severity? ii. How does the COVID-19 disease course on go for FMF patients who have been taking colchicine as a medication over the years after getting infected with SARS-CoV-2? iii. Is there an initial gut micribiota structure pattern for non-FMF and FMF patients in the cases when COVID-19 appears in mild form?

**Methods:**

The gut microbiota composition in non-FMF and FMF patients before the first infection (mild and moderate course of COVID-19) was considered. COVID-19 was diagnosed by SARS-CoV-2 nucleic acid RT-PCR in nasopharyngeal swab and/or sputum.

**Results:**

The number of patients with male FMF with mild COVID-19 was approximately two times higher than that of non-FMF male subjects with COVID-19. In addition, an association of COVID-19 disease severity with the baseline gut *Prevotella, Clostridium hiranonis, Eubacterium biforme, Veillonellaceae, Coprococcus*, and *Blautia* diversities in the non-FMF and FMF populations were revealed by us, which can be used as risk/prognostic factor for the severity of COVID-19.

## Introduction

The causative agent of COVID-19, the SARS-CoV-2 virus, like all other viruses, changes over time. The changes in virus properties may be unnoticeable but are likely to affect the rate at which the virus spreads, the course of the disease, the severity, the diagnosis, the prevention, the effectiveness of the vaccination, and the treatment. Conventionally, according to the progress of COVID-19, the disease is divided into four variants: mild (without chest imaging findings and mild clinical symptoms), moderate (with chest imaging presenting mild pneumonia manifestation), severe (with chest imaging showing the lesions significantly progressed), and critical (rapid progression of the disease) (Gao et al., 2021).

The emergence of SARS-CoV-2 variants in late 2020 caused great concern in the international arena, forcing the World Health Organization (WHO) to prioritize the characterization and differentiation of virus variants. One of the designated variants of concern is alpha, beta, gamma, delta, or omicron (Layton and Sadria, 2022).

Literature on SARS-CoV-2-alpha, -beta (Guan et al., 2020; Hussain et al., 2020; Kossumov et al., 2021), -gamma and -delta variants indicate a link between the severity of COVID-19 and diabetes (Bachache et al., 2022). Moreover, the majority of patients with COVID-19 are prone to impaired glucose metabolism, which emphasizes the importance of controlling the glucose metabolism of patients even if they have not had problems with it before (Wang et al., 2020; Bachache et al., 2022; Yonekawa and Shimono, 2022). According to Hu and co-authors, there was no age–sex difference between the delta-type and wild-type groups in patients with COVID-19 (mean age 53.0 years). There was no difference in comorbidities, although delta patients had a reduced time interval between disease onset to hospitalization (Hu et al., 2022). Moreover, hypertension (Hussain et al., 2020; Schiffrin et al., 2020), acute coronary syndrome (Metzler et al., 2020), rheumatic (Misra et al., 2020), and gastrointestinal (Tsaturyan et al., 2022a) and neurological features (Manji et al., 2020; Mao et al., 2020) in SARS-CoV-2 infectivity have been reported (Tsaturyan et al., 2022a) to be in association with the changes in gut microbiota composition.

On the other hand, the associations between host physiology and gut bacteria (Khan et al., 2019; Ragonnaud and Biragyn, 2021; Tsaturyan et al., 2022b), including host blood characteristics and gut bacteria (Pluznick, 2014; Balayan et al., 2015; Pepoyan et al., 2015a, 2020c), gut microbiota composition, and COVID-19-accompanying diseases as well as between host gender and microbiota composition are at the researchers' attention (Pepoyan et al., 2021). *Faecalibacterium prausnitzii, Eubacterium rectale*, and Bifidobacteria, the gut commensals with known immunomodulatory potential, were described to be underrepresented in patients with COVID-19. Moreover, these bacteria continued to be low up to 30 days after disease resolution (Yeoh et al., 2021).

Familial Mediterranean fever (FMF), a monogenic autosomal recessive autoinflammatory disease (Touitou and Pepoyan, 2008; Almeida de Jesus and Goldbach-Mansky, 2013; Pepoyan et al., 2015b) has a very high incidence in Armenia (Pepoyan et al., 2017, 2021). According to our research data from the previous investigations on FMF, M694V/V726A pyrin inflammasome mutations leading to FMF may express gender-specific differences in these patients (Pepoyan et al., 2018a). Interestingly, despite varying literature, the beneficial impact of colchicine [the main medication for patients with FMF Terreri et al., 2016; Pepoyan et al., 2021], on the severity of COVID-19, is still debated (Recovery Collaborative Group, 2021; Tardif et al., 2022; Zein and Raffaello, 2022). For example, electronic databases such as PubMed, Google Scholar, and Cochrane were systematically collected until June 2021 by Yasmin et al. (2022) Based on a meta-analysis of a total population of 16,048 individuals, the authors concluded that colchicine reduced the overall severity of COVID-19 disease.

The investigation in this study applies to the process of COVID-19 disease among Armenians. The study has the purpose of answering three core questions: (i) Do the characteristics of the gut microbiome of Armenians affect the course of COVID-19 severity? (ii) How does the COVID-19 disease course emerge for patients with FMF who have been taking colchicine as a medication over the years after getting infected with SARS-CoV-2? and (iii) Is there an initial gut microbiota structure pattern for non-FMF and FMF patients in the cases when COVID-19 appears in a mild form?

Hence, the investigations in this study aimed to evaluate the associations between the baseline gut microbiota composition of healthy and FMF-carrier Armenian male populations, and the severity of the COVID-19 disease after their infection with the SARS-CoV-2.

Considering the fact that according to the National Center for Disease Control and Prevention, the alpha-, beta- and delta- variants of the SARS-CoV-2 were found in Armenia by 8 January 2022, this study refers to the comparative characterization of the gut microbiota composition in the non-FMF and FMF patients before the SARS-CoV-2 infection by September 2021. The gut microbiota composition in non-FMF and FMF patients before the first infection (mild, and moderate course of COVID-19) was considered.

## Materials and methods

The fact that gut microbiota composition is determined by the host's genetics (Khachatryan et al., 2008; Pepoyan et al., 2018b; Krainer et al., 2020; Bubier et al., 2021), and the fact that one of the advantages of the PhyloChip^TM^ microarray is the timelessness of the data concerning the latter, provided the basis for assessing the initial composition of gut microbiota of patients with COVID-19.

Taking into account the abovementioned facts, we re-examined/rediscussed/compared the previously obtained data by the PhyloChip^TM^ microarray (accession number GEO GSE111835^1^ (Pepoyan et al., 2015a, 2018a, 2021) on the composition of the gut microbiota of patients with FMF depending on the mild and moderate course of COVID-19.

The rates of SARS-CoV-2 infection and COVID-19 severity were evaluated for 20 healthy male volunteers and 20 healthy female volunteers (non-FMF participants), and 23 male volunteers and 22 female FMF volunteers (18–50 years), who were the participants in a previous trial as well, on gut microbiota investigation in FMF. After elucidating the course of COVID-19 in registered participants, the composition of the gut microbiota of patients with mild and moderate COVID-19 was compared. The comparisons of gut microbiota of “mild–mild” and “mild–moderate” subjects have given the opportunity to neutralize the flaws resulting from the insufficiency of patients in several categories.

To increase the number of non-FMF and FMF participants (both men and women) for the clarification of the COVID-19 severity rates among men and women, besides the trial participants, another 80 “non-FMF” individuals without mutations in the *MEFV* gene and 84 patients with FMF were randomly selected from different Armenian families. A total of 84 participants with FMF were chosen out of 37 families. In contrast with the trial, when we had a certain informational basis concerning the subjects' gut microbiota, no investigation of gut microbiota was held for these patients. Consequently, the contents of their gut microbiota were not examined.

A detailed explanation of the trial is given by Pepoyan et al. (2015b, 2018b, 2021). All the diagnoses of the patients were authenticated by genetic analysis. In the month prior to the investigation, every participant excluded any possible medication from their routine that could possibly modify the results of the study, such as hormones, chemotherapeutic agents, antibiotics, or probiotics. Everyone constantly ingested colchicine for 7 or more years with a daily dose of 1 mg. All the patients were asked to deliver their fecal materials to the laboratory in 2 h after collection. We used the UltraClean® Tissue and Cells DNA Isolation Kit (QIAGEN, Germantown, MD) and the ZR Fecal DNA MiniPrep (Zymo Research, Irvine, CA) to isolate the DNA by following the provided recommendations.

The primer sequences used for microarrays and 16S rRNA clone libraries were 27f.jgi (bacteria-specific) 5′-AGAGTTTGATCCTGGCTCAG-3′ and 1492r.jgi (Bacteria/Archaea-specific) 5′-GGTTACCTTGTTACGACTT-3′. The bacterial communities discovered in the feces were evaluated by a third-generation, culture-independent, high-density DNA microarray (PhyloChip^TM^; Affymetrix, Santa Clara, CA) analysis as it was mentioned before (Kellogg et al., 2013). This outlook observes and measures the correlative amplitude of ~50,000 discrete microbial taxa. The latter revolves around the analysis of the chain of 16S ribosomal RNA genes. The PhyloChip^TM^ relies on the analysis of every variable region of the 16S gene, offering a more extensive taxonomic classification than any other method. The latter method, with around 1.2 million probes per chip, guarantees that the measurements on ascendant bacteria do not extinguish the fundamental low-abundance ones.

Roughly, full-length 16S rRNA-gene particles were boosted with the use of bacterial primers. The PhyloChip^TM^ analysis utilized amplicons, evaluating the differences in hybridization intensity—reflective of differences in the relative abundance of bacterial taxa (Kellogg et al., 2013).

COVID-19 was diagnosed using SARS-CoV-2 nucleic acid RT-PCR in nasopharyngeal swabs and/or sputum. The severity rate of COVID-19 disease was described by chest imaging findings: patients with the mild form of COVID-19 had mild clinical symptoms over the disease period with no chest imaging findings, while the patients with the moderate form carried mild pneumonia manifestation in their chest imaging, and the critical form defined a rapid progression of the disease.

The Mann–Whitney statistical analysis and the *t*-test were performed to test the null hypothesis in Microsoft Excel 2016.

## Results

### COVID-19 severity rates among Armenian non-FMF- and FMF-diseased male and female populations

According to the investigations from this study, COVID-19 severity rates were lower in participants with FMF compared with the same rate for non-FMF volunteers.

None of the 22 investigated FMF male patients bore the severe form of COVID-19, 19 of them had the mild form of the disease, and only 3 of them experienced the moderate form (Table 1). In contrast, 2 out of 20 non-FMF participants of the trial had the severe form of COVID-19, 8 experienced the mild form and 10 underwent the moderate course of the disease.

**Table 1:** COVID-19 severity rates among Armenian male non-FMF and FMF^*^ diseased populations.

**Subjects**	**Number of COVID-19 subjects with mild form**			**Number of COVID-19 subjects with moderate form**			**Number of COVID-19 subjects with severe form**		
	**All**	**Trial** ^**^	**None** ^***^ **trial**	**All**	**Trial**	**None trial**	**All**	**Trial**	**None trial**
Non-FMF	24	8	16	30	10	20	6	2	4
FMF	55	19	36	9	3	6	0	0	0

* Patients with familial Mediterranean fever disease.

** Trial participants.

*** None of the trial participants.

Outside the trial, 36 out of 42 FMF subjects had the mild form of COVID-19; 16 out of 40 non-FMF patients had the same experience with the disease, whereas 20 had the moderate form.

Overall, out of 126 male patients, 79 had the mild form of COVID-19 (55 FMF and 24 non-FMF patients) (2.3:1). That is, the number of male FMF patients with mild COVID-19 was approximately two times higher than the non-FMF male representatives with COVID-19. This number was lower for women with FMF than for women without it. Out of 125 investigated female participants, 96 had COVID-19 in a mild form (44 FMF and 52 non-FMF subjects).

### Gut microbiota composition in the population of Armenian men without FMF

**Figure 1:**
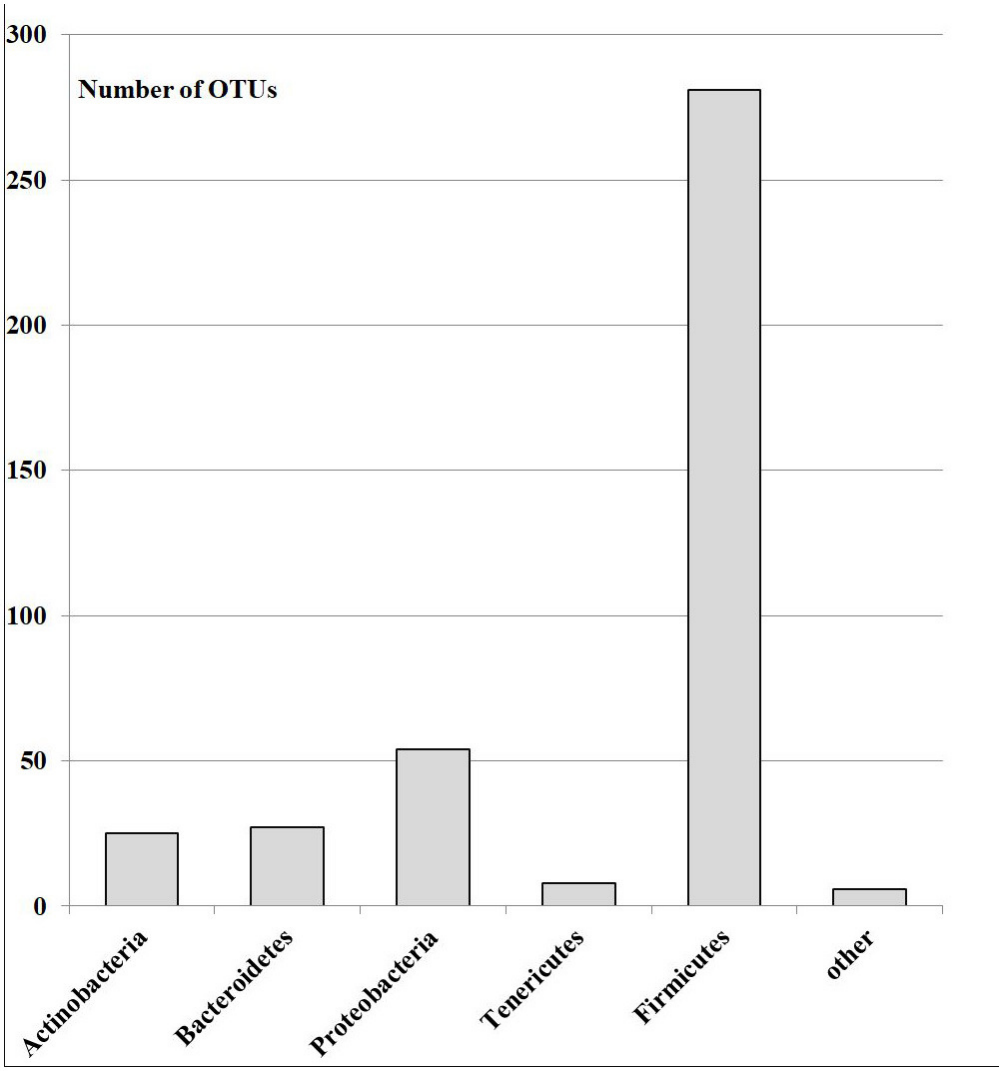
The number of OTUs of the main different bacterial phylum in the gut microbiota of patients with mild and moderate COVID-19 (healthy non-FMF male subjects before the infection with SARS-CoV-2); *p* < 0.05.

A total of 18,700 bacterial operational taxonomic units (OTUs) were identified for the investigated Armenian population without FMF. Approximately 400 OTUs from those were statistically different between men having COVID-19 in mild and moderate forms (*p* < 0.05). Interestingly, 70.07% of these OTUs belonged to *Firmicutes*, ~13.73% belonged to *Proteobacteria*, 2% belonged to *Tenericutes*, 6.75% belonged to *Bacteroidetes*, 6.23% belonged to *Actinobacteria*, and 1.5% belonged to different phyla, which represented <3 OTUs (Figure 1).

#### Firmicutes

**Figure 2:**
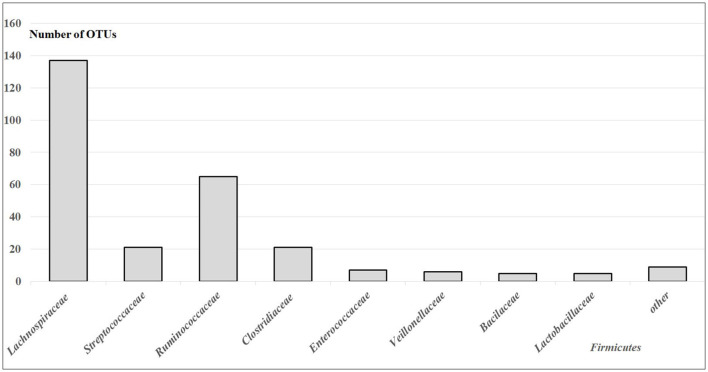
The number of OTUs of main different gut *Firmicutes* in the gut microbiota of patients with mild and moderate COVID-19 (healthy non-FMF male subjects before the infection with SARS-CoV-2); *p* < 0.05.

Within *Firmicutes*, prevailed differences were related to OTUs of the families *Lachnospiraceae*-−49.64%, *Ruminococcaceae*-−23.55%, *Streptococcaceae*-−7.61%, *Clostridiaceae*-−7.61%, *Enterococcaceae*-−2.54, *Veillonellaceae*-−2.17%, *Bacilaceae*-−1.81%, *Lactobacillaceae—*1.81%, and 3.26% related to other families with <5 OTUs (Figure 2). The differences in gut *Lachnospiraceae* baseline levels of COVID-19 male patients in the mild and moderate forms are presented in Table 2. The bacterial concentrations (are correlated with the hybridization scores) of *Faecalibacterium* spp., *Coprococcus* spp., *Blautia* spp., and *Eubacterium* spp. were higher in the mild form of the disease, while the concentrations of *Enterococcus* spp. were higher in the moderate form of the disease (*p* < 0.05).

**Table 2:** Hybridization scores for the different gut *Lachnospiraceae* at baseline microbiota of COVID-19 subjects in mild and moderate forms, *p* < 0.05.

**Bacteria**	**Number (different OTUs)**	**Hybridization scores**	
		**Mild COVID-19**	**Moderate COVID-19**
*Faecalibacterium* spp.	39 (28.47%)	495,677.68	435,823.06
*Coprococcus* spp.	25 (18.25%)	163,437.83	116,112
*Blautia* spp.	18 (13.14%)	90,964.87	60,926
*Eubacterium* spp.	12 (8.76%)	92,104.51	61,718
*Enteroccocus* spp.	7 (5.11%)	16,080.58	24,723

Percentage of OTUs within the family is given in parentheses.

**Figure 3:**
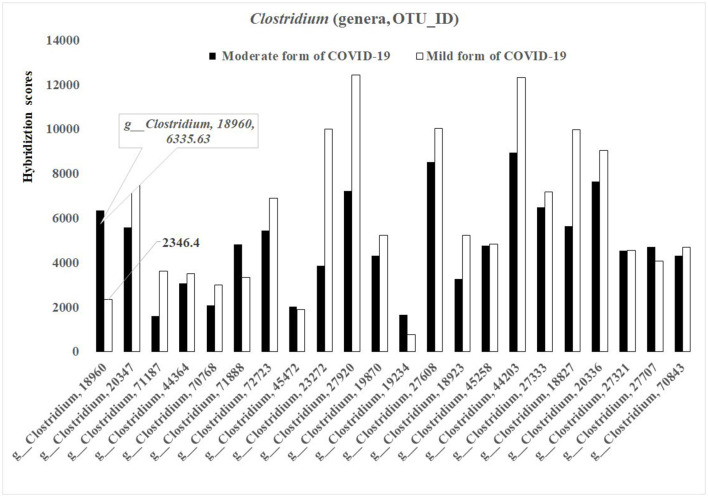
Bacterial differences in the baseline gut microbiota composition of COVID-19 male patients in mild and moderate form (genus *Clostridium*; *p* < 0.05).

In general, the concentrations of the different bacterial species from the genus *Clostridium* [number of different OTUs-−27 (19.71%)], were higher in the baseline gut microbiota of the mild form of COVID-19 patients than that in the moderate form (Figure 3). However, for several species (for example, OTU_ID 18960), the concentration was lower in baseline gut microbiota in mild diseased patients in comparison with those of the moderate form (2 346.4 vs. 6 335.63; *p* < 0.05) (Figure 3).

#### Proteobacteria

**Figure 4:**
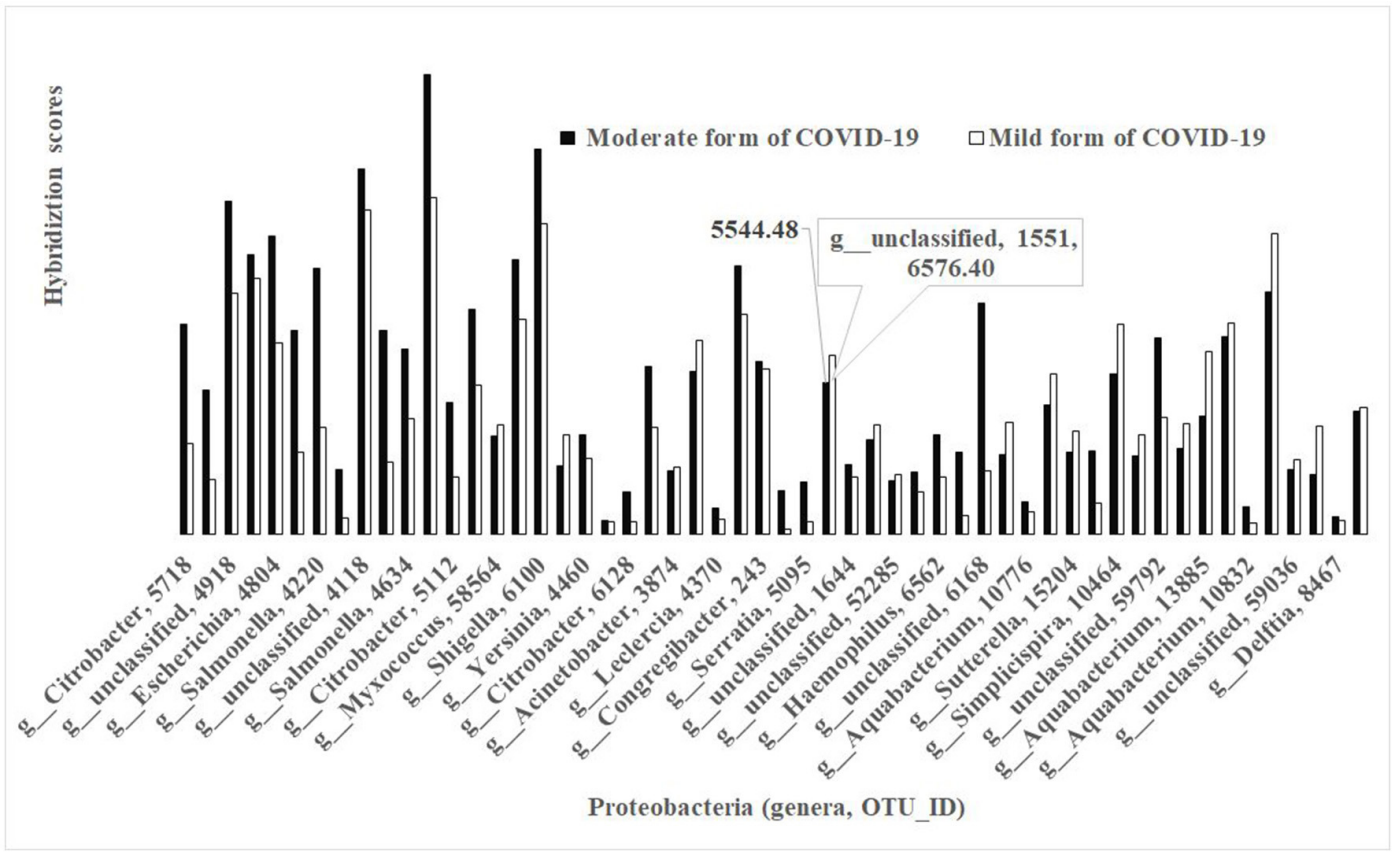
Bacterial differences in the baseline gut microbiota composition of COVID-19 male patients in mild and moderate form (phylum *Proteobacteria; p* < 0.05).

The phylum *Proteobacteria* represented 59 OTUs in different bacteria between the non-FMF mild and moderate groups (*p* < 0.05) and carried a maximum of 5 OTUs from the same genus. The concentrations of the bacterial species were lower in the baseline gut microbiota of the mild form of COVID-19 patients than of those with the moderate form (Figure 4). Only a very limited number of species from different genera, including, for example, unclassified OTU_ID 1551 (Figure 4), had a comparatively high concentration in baseline gut microbiota in mild diseased patients in comparison with those of the moderate form (6 576.40 vs. 5 544.48; *p* < 0.05) (Figure 4).

#### Actinobacteria

Most of all statistically different *Actinobacteria* related to the family *Corynebacteriaceae* (80.00%), and only a species of *Bifidobacterium longum* was different from the order *Bifidobacteriales*.

#### Bacteroidetes

Within *Bacteroidetes* prevailed differences related to *Prevotella* spp. In general, the concentrations of *Prevotella* spp. were lower in the baseline gut microbiota of men, which carried a moderate form of COVID-19 after the infection with the SARS-CoV-2 (88,852.72 vs. 154,966.51; *p* < 0.05).

#### Comparative gut microbiota composition analysis between the non-FMF and FMF men, the people who carried mild COVID-19 disease after their infection with the SARS-CoV-2

**Figure 5:**
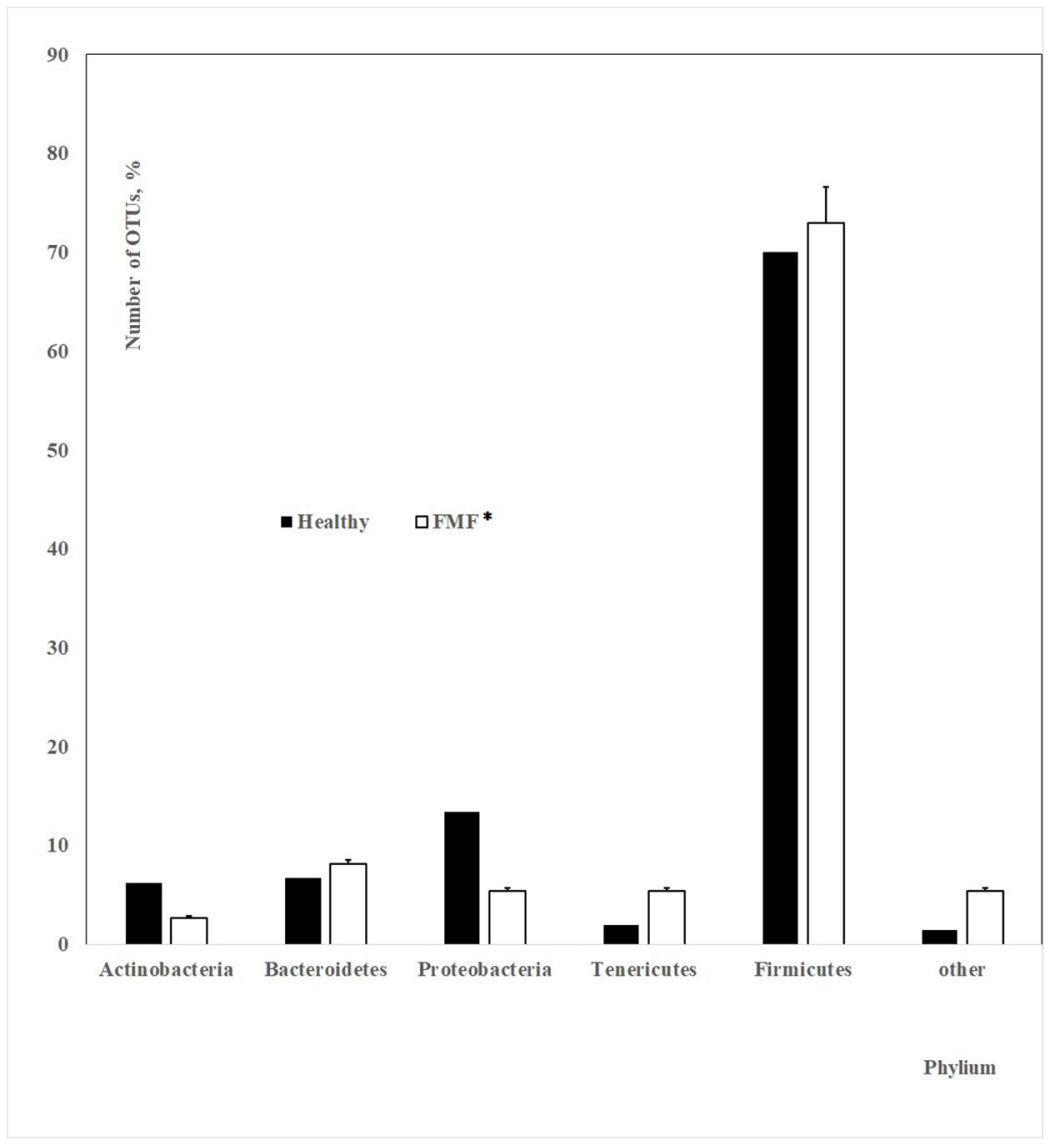
The number of OTUs of main different bacterial phylum in the gut microbiota of patients with mild and moderate COVID-19 (healthy non-FMF/FMF* male subjects before the infection with the SARS-CoV-2; in percentage, *p* < 0.05). ^*^Patients with familial Mediterranean fever disease.

During the investigations in this study, the baseline levels of gut bacteria in mild and moderate COVI-19-diseased men with FMF were compared to men without FMF (Figure 5). Statistically significant differences between mild non-FMF and moderate non-FMF; mild FMF and moderate non-FMF; and mild non-FMF and moderate FMF patients' gut microbiota (*p* < 0.05) as well as the similarity between mild–mild non-FMF/FMF and moderate–moderate non-FMF/FMF patients' gut microbiota (*p* > 0.05) allowed us to distinguish 74 important OTUs belonged to the following families:

- Family *Clostridiaceae* (six OTUs, two of which are related to *Clostridium hiranonis*);
- Family *Erysipelotrichaceae* (three OTUs, two of which are related to *Eubacterium biforme);*
- Family *Lachnospiraceae* (34 OTUs);
- Family *Lactobacillaceae* (five OTUs, an OTU related to *Lactobacillus agilis*);
- Family *Prevotellaceae* (five OTUs);
- Family *Ruminococcaceae* (three OTUs);
- Family *Veillonellaceae* (three OTUs);
- Unclassified (4 OTUs), and 11 OTUs from the 74 OTUs related to any other bacterial family.

Within *Lachnospiraceae*, the prevailed OTUs that met the aforementioned requirements were as follows:

- Genus *Blautia* (four OTUs).
- Genus *Clostridium* (four OTUs).
- Genus *Coprococcus* (four OTUs).

## Discussion

To check the main hypothesis if the baseline gut microbiota composition plays an important role in the severity of COVID-19 disease and if there might be a “common” bacteria, which is important for the severity of COVID-19 disease in non-FMF and FMF populations, we aimed to investigate (i) COVID-19 severity rates among Armenian non-FMF- and FMF-diseased populations and (ii) initial gut microbiota composition in Armenian non-FMF- and FMF-diseased male populations that carried COVID-19 in mild and moderate forms. Unfortunately, there were no data on the composition of the gut microbiota of colchicine-naïve FMF patients and the incidence of COVID-19 in these patients to assess the impact of colchicine on COVID-19.

### COVID-19 severity rates among Armenian non-FMF- and FMF-diseased male and female populations

According to the investigations, the severity and mortality rate of COVID-19 infection was higher among men than among women (Fisher and Ryan, 2021) which might be explained by the sex differences in immune response between men and women (Klein et al., 2020). Sex differences in incidence and mortality have been found in many infectious diseases. For COVID-19 disease, factors such as sex differences in the prevalence of underlying diseases may play a part in the case–fatality rate (CFR) differences. However, the consistently greater CFR in male populations of all ages suggests the impact of sex-related factors on the natural history of COVID-19 (Green et al., 2021). Both literature and analyses of this study have shown that the severity and mortality rate of COVID-19 infection is higher in men than in women. We hypothesized that the low severity rates of COVID-19 in patients with FMF, which were associated with their sex, could be also explained by the impact of colchicine and alterations in the gut microbiota of patients.

### Initial gut microbiota composition

The gut microbiome, which collected ~38 trillion microbes, has an important impact on host health (Sender et al., 2016). The composition of the gut microbiome depends on ethnicity (Lewis et al., 2017; Louis-Jean and Martirosyan, 2019), environmental and lifestyle factors (Bowyer et al., 2019; Koliada et al., 2020), as well as host age (Badal et al., 2020). According to Yeoh et al., the differences were found in several gut bacterial diversities of the phyla *Bacteroidetes* and *Actinobacteria* between the non-COVID-19 non-FMF people and patients with COVID-19; representatives of the *Bacteroidetes* were described to be more abundant and *Actinobacteria* were described to be less in patients with COVID-19 (Yeoh et al., 2021). Furthermore, recently, not only the gut commensals with known immunomodulatory potential described to be underrepresented in patients with COVID-19 but also COVID-19 severity in association with population-level gut microbiome variations was discussed (Lymberopoulos et al., 2022). These results, in parallel with other studies, highlight the potential utility of multi-kingdom host phenotype and microbiota profiling as a predictive tool for patients with COVID-19 (Liu et al., 2022). While the role of the gut microbiota in the course of COVID-19 infection in healthy populations is becoming increasingly clear, similar studies are lacking in diseased populations, including patients with FMF.

Our investigations emphasized the initial gut state of patients with COVID-19 and the severity of COVID-19 disease, which showed differences in ~400 initial bacterial OTUs (prevailed number of OTUs related to the family *Lachnospiraceae*; phylum *Firmicutes*) between men having COVID-19 in mild and moderate forms (*p* < 0.05). In general, the concentrations of *Prevotella* spp. from the more abundant *Bacteroidetes* genus were lower in the baseline gut microbiota of men, which carried a moderate form of COVID-19 after the infection with the SARS-CoV-2 (88 852.72 vs. 154 966.51; *p* < 0.05). These relations on prevailed percentages in OTUs (Figure 1) and bacterial concentrations stayed relatively stable for the FMF patients with COVID-19 (Figure 5) despite the decrease of the numbers of OTUs in the principal gut bacteria in a more detailed comparison of the initial composition of gut microbiota in patients with mild and moderate COVID-19 (74 OTUs vs. 401 OTUs).

According to a number of studies, SARS-CoV-2 has an impact on the daily psychological and physical health of women. During the early stages of quarantine, women in Spain and Hungary showed more depression and anxiety than men (Ausin et al., 2020; Szabo et al., 2020). A similar pattern has been discovered in Israel after the initial lockdown (Horesh et al., 2020). According to our previous investigations on the comparative distribution of gut *Prevotella* in non-FMF men and women, the non-FMF female cohort had a lower abundance of *Prevotella* in comparison with non-FMF men (*p* < 0.05) (Pepoyan et al., 2021), which might affect the comparative infectivity of SARS-CoV-2 and COVID-19 severity rates of non-FMF and FMF populations.

To propose specific gut microbiota markers in association with improved immune response and reduced adverse events following COVID-19 vaccines, Ng and co-authors investigated the gut microbiota composition in association with the SARS-CoV-2 vaccine (Ng et al., 2022). *Bifidobacterium adolescentis, Roseburia feces, Prevotella copri*, and *Megamonas* spp. were chosen by these authors as microbiota-targeted markers to increase the effectiveness in screening COVID-19 vaccines (Ng et al., 2022). According to our investigations in this study, both the concentrations and the abundance of specific gut strains (*Faecalibacterium* spp., *Coprococcus* spp., *Blautia* spp., *Eubacterium* spp., and *C. hiranonis*) might be informative in the diagnosis of the COVID-19 severity and its prevention approaches. Analysis of the relative concentrations/abundances of main phyla/families/genera in baseline gut microbiota may be the basis for the development of COVID-19 disease severity diagnosis approaches and future suggestions of appropriate preventive techniques through immunostimulants and vaccines. In addition, taking into account the fact that probiotics have beneficial effects on the host's health (Reid, 2005; Galstyan et al., 2018; Pepoyan et al., 2018b, 2020a,b; Harutyunyan et al., 2022), and are already suggested in COVID-19 treatments (Nguyen et al., 2022; Suvorov et al., 2022), the findings of this study will help during corrective probiotic therapies when applied to COVID-19 cases.

Meanwhile, the key role of colchicine in NLPR3 inflammasome-related processes is also discussed, resulting in IL-6-mediated reduction of IL-6 synthesis and CRP (Pepoyan et al., 2018a; Parra-Medina et al., 2020). Although data on the efficacy of colchicine in COVID-19 are limited and controversial, according to a meta-analysis by Yasmin et al., taking colchicine reduces the overall severity of COVID-19 disease (Yasmin et al., 2022). Future opportunities in colchicine-naïve FMF patients will also allow us to determine the hypothetical effect of colchicine on COVID-19.

More detailed studies on the initial microbiota composition are needed to avoid possible inaccuracies coming from outdated information on culture-independent, high-density DNA microarray analysis, previously assessed by a third generation. On the contrary, one of the advantages of the microarray is the timelessness of the data concerning the latter. The new developments on already existing data on DNA microarray might be important for inceptive decisions concerning orientations/fighting against unknown infections because of the difficulties to organize trials from the point of view of biosafety and time deficiency.

## Conclusion

The following conclusions might be pointed out from the investigations of this study:

- Both SARS-CoV-2 infection and COVID-19 severity rates are lower in the Armenian FMF-diseased population compared with the same rate for non-FMF population.
- There is an association between initial gut microbiota composition and COVID-19 disease severity in non-FMF and FMF populations.

Finally, there is an association between the baseline gut *Prevotella, C. hiranonis, E. biforme, Veillonellaceae, Coprococcus*, and *Blautia* diversities in non-FMF and FMF populations, and COVID-19 disease severity; the changes in diversities/concentrations of these bacteria might be used as risk/prognostic factors for the severity of COVID-19.

## Data availability statement

The datasets presented in this study can be found in online repositories. The names of the repository/repositories and accession number(s) can be found in the article/supplementary material.

## Ethics statement

The studies involving human participants were reviewed and approved by the Ethics Committee at the Ministry of Education and Science of Armenia. Also, all investigated participants gave written informed consent prior to the study (Pepoyan et al., 2018b).

## Author contributions

AP and VT contributed to the conception and design of the study. AP wrote the first draft of the manuscript. VT, TT, MC, EP, and NH wrote sections of the manuscript. AM, MB, and VT contributed experimental data. All authors read, revised, and approved the submitted version.

## Footnotes

^1^ https://www.ncbi.nlm.nih.gov/geo/query/acc.cgi?acc=GSE111835.s
